# Supplementary material for: Magnetic crystalline-symmetry-protected axion electrodynamics and field-tunable unpinned Dirac cones in EuIn2As2
Source: Nat Commun. 2021 Feb 12;12:999. doi: 10.1038/s41467-021-21154-y (PMC7881193; doi:10.1038/s41467-021-21154-y)
Supplement: Supplementary file 1 — Supplementary Information [file 41467_2021_21154_MOESM1_ESM.pdf]

Magnetic crystalline-symmetry-protected axion electrodynamics and field-tunable unpinned Dirac cones in $EuIn_2As_2$

(Supplementary Information)

S. X. M. Riberolles,[1] T. V. Trevisan,[1,2] B. Kuthanazhi,[1,2] T. W. Heitmann,[3] F. Ye,[4] D. C. Johnston,[1,2] S. L. Bud'ko,[1,2] D. H. Ryan,[5] P. C. Canfield,[1,2] A. Kreyssig,[1,2] A. Vishwanath,[6] R. J. McQueeney,[1,2] L. L. Wang,[1,2] P. P. Orth,[1,2] and B. G. Ueland[1]

[1]*Ames Laboratory, Ames, IA, 50011, USA*

[2]*Department of Physics and Astronomy, Iowa State University, Ames, IA, 50011, USA*

[3]*University of Missouri Research Reactor, Columbia, MO, 65211, USA*

[4]*Oak Ridge National Laboratory, Oak Ridge, TN, 37830, USA*

[5]*Physics Department and Centre for the Physics of Materials, McGill University, Montreal, Quebec H3A 2T8, Canada*

[6]*Department of Physics and Astronomy, Harvard University, Cambridge, MA, 02138, USA*

CONTENTS

Supplementary Note 1. Single-crystal neutron diffraction experiments 2

Supplementary Note 2. Magnetic susceptibility, magnetization, and resistance 11

Supplementary Note 3. Details of the ^{151}Eu Mössbauer study 15

Supplementary Note 4. Additional density functional theory results 19

Supplementary Note 5. Symmetry analysis of the magnetic phases 21

References 30

Supplementary Note 1. SINGLE-CRYSTAL NEUTRON DIFFRACTION EXPERIMENTS

In the following, we detail our determination of the $T < T_{\mathbf{N2}}$ magnetic order using the TRIAX and CORELLI data.

We first detail the symmetry analysis and magnetic space group (MSG) determination procedures which utilized the Bilbao Crystallography Server [1] and simulations of diffraction patterns made with MAG2POL [2]. Combinations of two distinct magnetic phases individually corresponding to $\boldsymbol{\tau}_1$ [approximated to $\boldsymbol{\tau_1} = (0, 0, \frac{1}{3})$] and $\boldsymbol{\tau}_2 = (0, 0, 1)$ were discarded as they returned spin-density-wave rather than local-moment type AF order. Local-moment type AF order is more accurate than itinerant AF order based on our band-structure calculations indicating that bands with significant Eu $4f$ character lie well below the Fermi energy E_{F}.

We next searched for a single MSG with symmetry elements leading to reflection conditions consistent with both $\boldsymbol{\tau}_1$ and $\boldsymbol{\tau}_2$ that is also a subgroup of the paramagnetic grey MSG $P6_3/mmc1'$ (No. 194.264). We found that MSGs $Cmcm$ (No. 63.457), $Cm'c'm$ (No. 63.452), $C2'2'2_1$ (No. 20.33), $C222_1$ (No. 20.31), and $P2_1/m$ (No. 11.50) are consistent with both propagation vectors. Note that additional MSGs are consistent with these propagation vectors but require further symmetry reductions and were thus disregarded. We ruled out $Cmcm$, $Cm'c'm$, $C222_1$ and $P2_1/m$ by simulating diffraction patterns for both $\boldsymbol{\mu} \perp \mathbf{c}$ and $\boldsymbol{\mu} \parallel \mathbf{c}$ and comparing them to $T = 6$ K data. Examples of simulated profiles are shown in

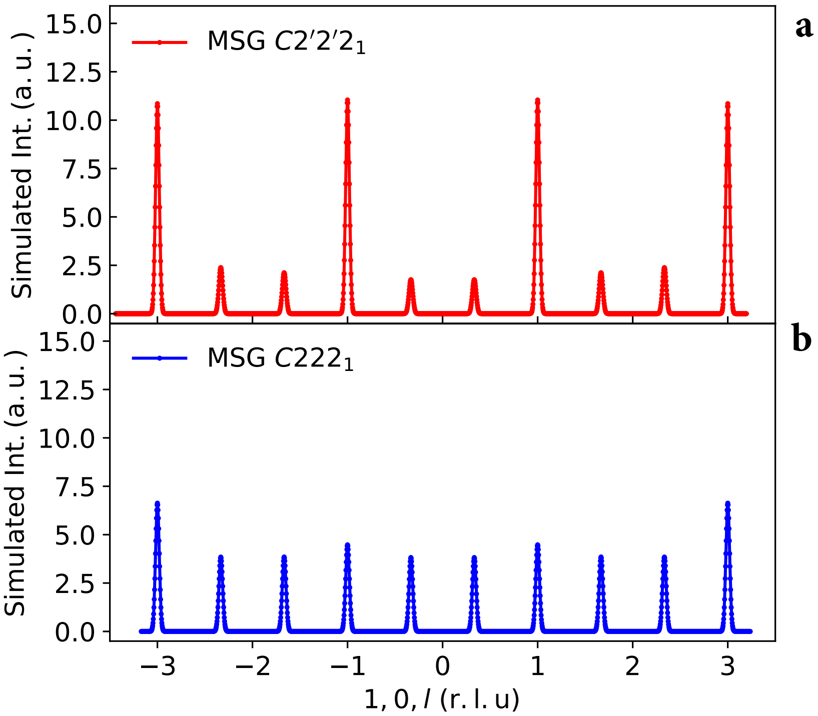

Supplementary Figure 1. **Simulated magnetic neutron diffraction patterns for broken-helix order with $\boldsymbol{\mu} \perp \mathbf{c}$. a,b**, Simulated pattern for magnetic space group (MSG) $C2'2'2_1$ (a) and MSG $C222_1$ (b) with helical-turn angles $\phi_{\rm rr} = -80°$, $\phi_{\rm rb} = 130°$, and $\mu = 6.0\ \mu_{\rm B}$.

Supplementary Figure 1 which can be compared to 6 K diffraction data in Supplementary Figure 2a. In particular, Supplementary Figure 1 illustrates the clear difference between the relative heights of commensurate and incommensurate magnetic Bragg peaks for $C2'2'2_1$ and $C222_1$, revealing the incompatibility of the latter with the 6 K data in Supplementary Figure 2a. We are thus left with $C2'2'2_1$ as the MSG for the $T < T_{\rm N2}$ AF order. Supplementary Table I shows the positions for the two Eu sites [referred to as Eu(red) and Eu(blue)] in $C2'2'2_1$ assuming a tripling along **c** of the hexagonal chemical-unit cell. Constraints imposed by the MSG on the orientation of the ordered magnetic moment associated with each Eu site are also indicated.

We next detail the steps of the refinements. Examples of TRIAX data not corrected for neutron absorption are shown in Supplementary Figs. 2b and 3. Supplementary Tables II and III list the Bragg peaks used for the refinements along with their integrated intensities and the corresponding absorption-corrected values. First, a single-crystal refinement to the absorption corrected $T = 30$ K data [corresponding to the paramagnetic (PM) phase] using

Supplementary Table I. **The Eu crystallographic sites in magnetic space group $C2'2'2_1$ (No. 20.33).** Positions are given in the setting of the parent space group $P6_3/mmc$ (No. 194) with lattice parameters $(a, b, 3c)$. m_i are components of the ordered magnetic moment $\boldsymbol{\mu}$. This table was created using the Bilbao Crystallography Server [1].

Atoms	Coordinates with Magnetic Moments	Multiplicity	m_x, m_y, m_z (μ_B/Eu)
Eu(red)	$(0, 0, 0 \mid m_x, m_y, m_z)(0, 0, \frac{1}{6} \mid m_y, m_x, m_z)$ $(0, 0, \frac{1}{2} \mid -m_x, -m_y, m_z)(0, 0, \frac{2}{3} \mid -m_y, -m_x, m_z)$	4	$m_x = 2.35$ $m_y = 6.69$ $m_z = 0.00$
Eu(blue)	$(0, 0, \frac{1}{3} \mid m_x, -m_x, m_z)(0, 0, \frac{5}{6} \mid -m_x, m_x, m_z)$	2	$m_x = -3.39$ $m_z = 0.00$

the known chemical structure of the material [3, 4] was performed allowing the scale factor and atomic positions to vary. The site occupations and thermal parameters could not be refined. The magnetic structure was then refined using the 6 K absorption-corrected data corresponding to the magnetic order. This refinement used the magnetic symmetry files created on the Bilbao crystallography server [1] for $C2'2'2_1$ and the parameters found from the refinement to the 30 K data. To account for the localized nature of the Eu^{2+} magnetic moments, a refinement constraint of equal values of the total ordered magnetic moment μ for both sites was used. Supplementary Figure 4 illustrates the refinement which returned a goodness-of-fit value of $R_F = 7.50$. The determined magnetic structure at 6 K is the broken-helix order diagrammed in Fig. 1d as well as Supplementary Figure 5 with turn angles of $\phi_{rr} = -80(2)^\circ$ and $\phi_{rb} = 130(1)^\circ$, and $\mu = 5.9(2)$ μ_B/Eu lying within the **ab** plane.

This result is confirmed by a refinement to $T = 6$ K data collected on the CORELLI instrument for a different sample. Data corresponding to a reduced incident neutron energy bandwidth centered at $E = 50$ meV (1.54 Å) and spanning 45–55 meV were used in order to accurately perform a correction for neutron absorption using MAG2POL [2]. The absorption correction and refinement procedures employed were similar to these previously described for the TRIAX data analysis. Sets of 18 nuclear (22 K) and 108 magnetic (6 K) independent reflections were used. We obtained turn angles of $\phi_{rr} = -68(2)^\circ$ and $\phi_{rb} = 124(1)^\circ$, $\mu =$

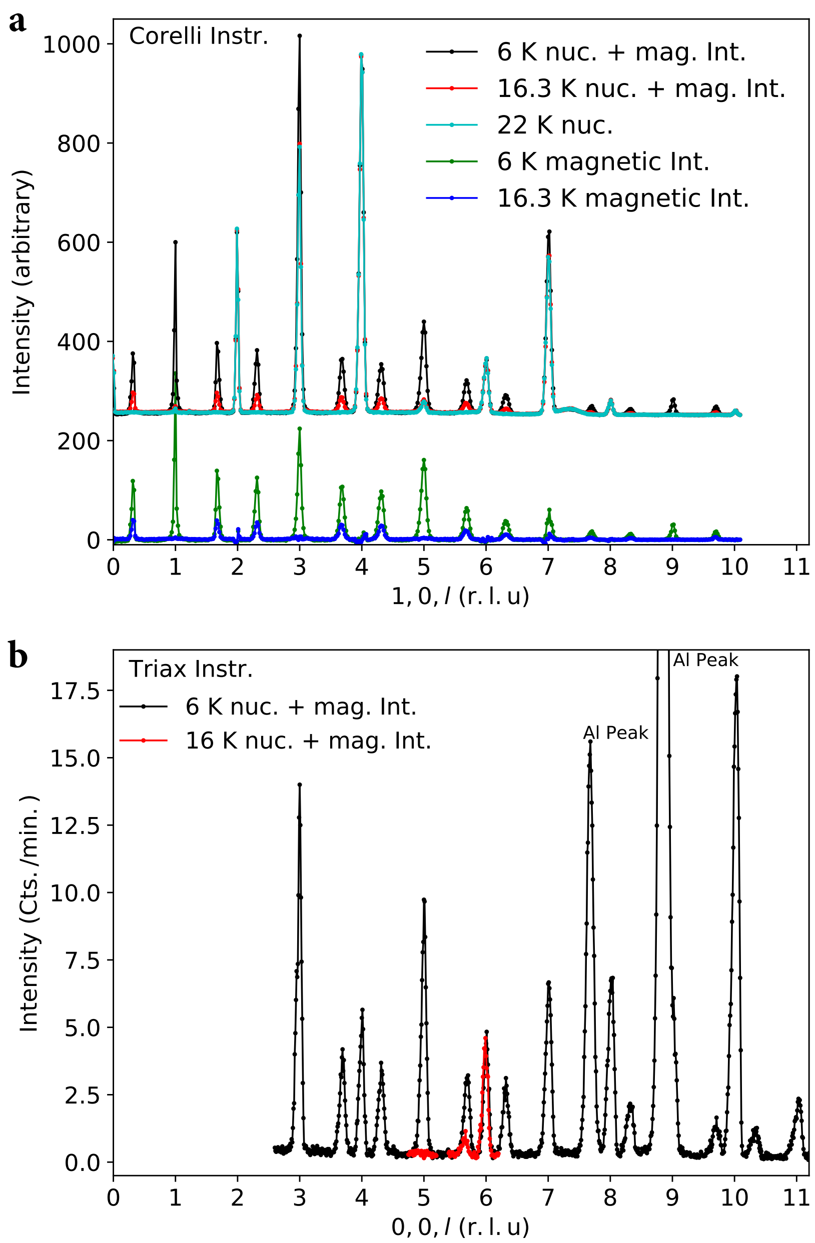

Supplementary Figure 2. **Diffraction patterns from measurements made on CORELLI and TRIAX.** **a**, Line cuts of CORELLI data along ($10l$) collected at T = 6 (black), 16.3 (red) and 22 K (cyan). For clarity, a 250 counts/minute offset has been applied. The 16.3 (blue) and 6 K (green) data not offset are the results of subtracting off the 22 K data in order to visualize the magnetic diffraction. **b**, ($00l$) line cut collected on TRIAX at 6 (black) and 16 K (red).

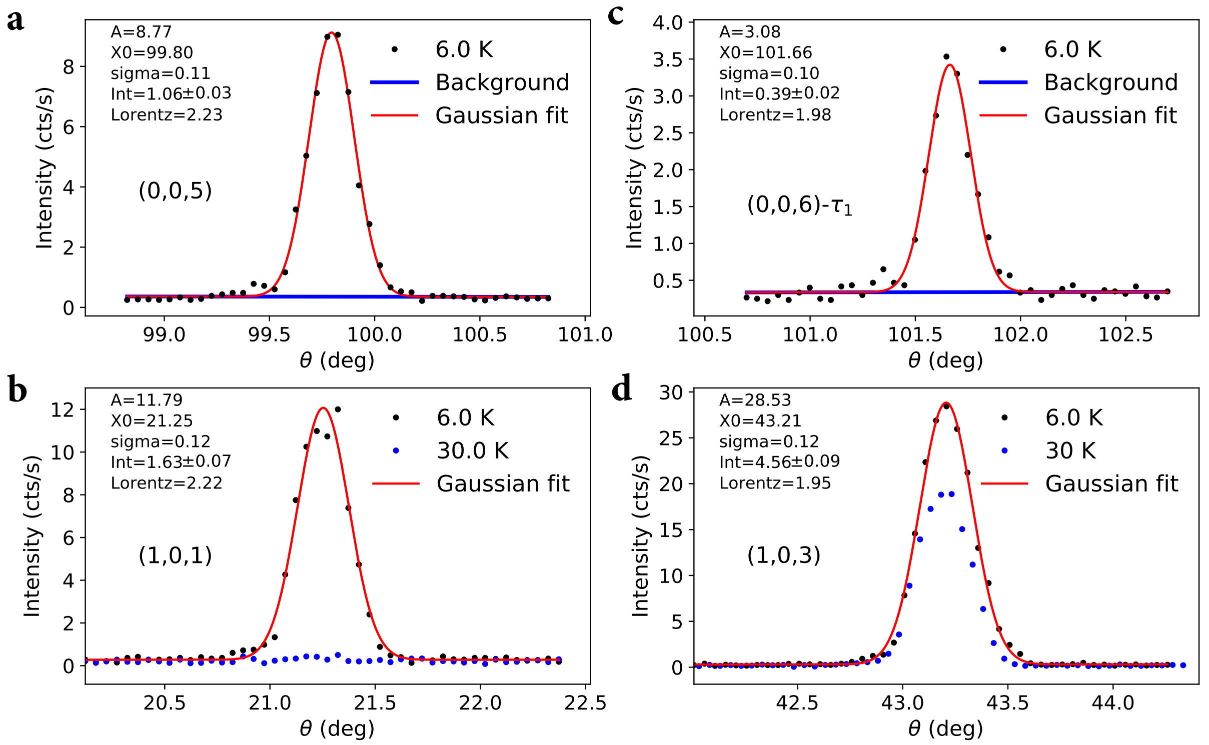

Supplementary Figure 3. **Examples of Bragg peaks collected on TRIAX. a–d**, Measured profiles for T = 6 and 30 K. The 30 K data are substituted by background lines for purely magnetic reflections in **a** and **c**. Each peak is fit using the combination of a Gaussian lineshape [y=$A\exp(-\frac{(x-x_0)^2)}{2\sigma^2})$] and a linear background. The obtained fit parameters and the corresponding integrated intensities corrected by the Lorentz factor (labeled as Int) are displayed in each panel.

6.1(2) μ_B/Eu and a goodness-of-fit value of $R_F = 17.2$. Figure 6 illustrates the quality of this refinement.

In the following, we detail our determination of the $T_{\mathbf{N2}}<T\leq T_{\mathbf{N1}}$ magnetic order using TRIAX and CORELLI data.

A reliable refinement of the TRIAX data for $T_{N2} < T \leq T_{N1}$ could not be completed. This is due to the rather weak magnetic intensities at these temperatures because of the smaller value of μ and the strong neutron absorption. Nevertheless, data in Fig. 2b and Supplementary Figure 2 reveal the systematic absence of Bragg peaks corresponding to $\boldsymbol{\tau}_2$ for $T > T_{N2}$. The presence of magnetic Bragg peaks at positions corresponding to $\boldsymbol{\tau}_1$ is in agreement with the stabilization of pure 60°-helix order and the reflection conditions for MSGs $P6_12'2'$ (No. 178.159), $P6_122$ (No. 178.155), $P6_52'2'$ (No. 179.165), and $P6_522$ (No.

Supplementary Table II. **Integrated intensities for Bragg peaks recorded on TRIAX at $T = 30$ K.** The correction for neutron absorption was applied using MAG2POL [2] as described in Ref. [5] .

$(h, 0, l)$	Integrated Intensity	Absorption Corr. Intensity
$(1, 0, 0)$	0.29 ± 0.02	0.41 ± 0.03
$(1, 0, 2)$	1.70 ± 0.05	2.47 ± 0.07
$(1, 0, 3)$	3.08 ± 0.06	4.65 ± 0.09
$(0, 0, 6)$	0.60 ± 0.03	1.53 ± 0.08
$(1, 0, 4)$	5.04 ± 0.21	8.08 ± 0.34
$(2, 0, 0)$	0.23 ± 0.02	0.33 ± 0.03
$(2, 0, 2)$	1.22 ± 0.03	1.79 ± 0.04
$(2, 0, 3)$	1.96 ± 0.03	2.93 ± 0.04
$(2, 0, 4)$	2.72 ± 0.10	4.17 ± 0.15

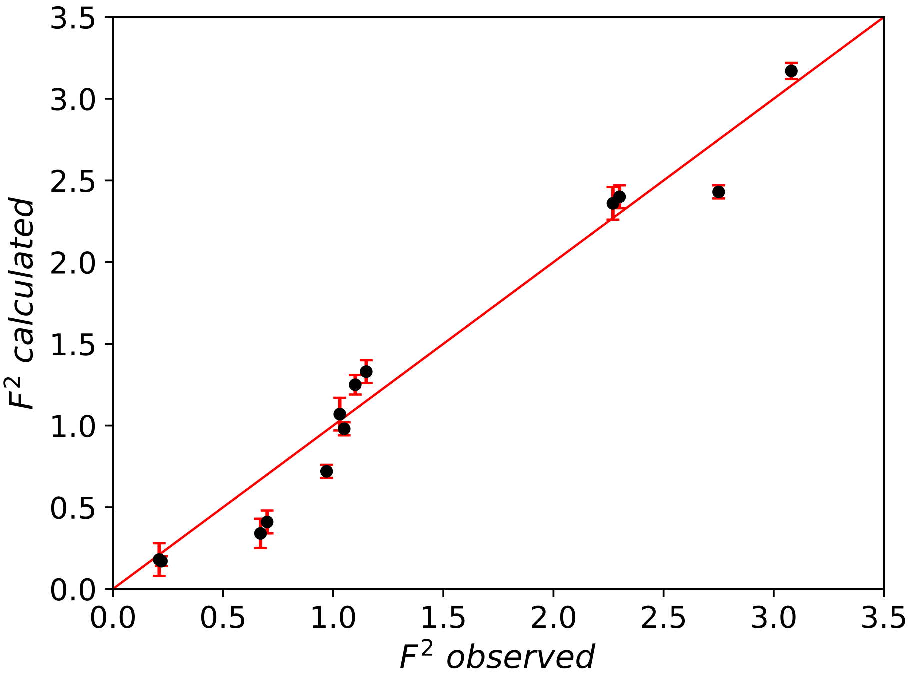

Supplementary Figure 4. **Calculated versus observed square of the magnetic structure factor F^2 for the refinement to data in Supplementary Table III using MSG $C2'2'2_1$.** The line indicates the refinement, which has a goodness of fit of $R_F = 7.5$.

Supplementary Table III. **Integrated intensities for Bragg peaks recorded on TRIAX at $T = 6$ K.** The correction for neutron absorption was applied using MAG2POL [2] as described in Ref. [5]. When present, nuclear contributions to the Bragg peaks were subtracted using the 30 K data given in Table. II.

$(h, 0, l)$	Integrated Intensity	Absorption Corr. Intensity
$(0,0,4) - \boldsymbol{\tau}_1$	0.31 ± 0.02	1.15 ± 0.07
$(0,0,4) + \boldsymbol{\tau}_1$	0.34 ± 0.02	1.10 ± 0.06
$(1,0,0) + \boldsymbol{\tau}_1$	0.48 ± 0.03	0.67 ± 0.04
$(0,0,5)$	1.06 ± 0.03	3.08 ± 0.09
$(1,0,1)$	1.63 ± 0.07	2.30 ± 0.10
$(1,0,2) - \boldsymbol{\tau}_1$	0.49 ± 0.03	0.70 ± 0.04
$(1,0,3)$	1.50 ± 0.07	2.27 ± 0.10
$(0,0,6) - \boldsymbol{\tau}_1$	0.39 ± 0.02	1.03 ± 0.05
$(0,0,6) + \boldsymbol{\tau}_1$	0.43 ± 0.03	1.05 ± 0.07
$(0,0,7)$	1.20 ± 0.03	2.75 ± 0.07
$(0,0,8) + \boldsymbol{\tau}_1$	0.47 ± 0.05	0.97 ± 0.10
$(2,0,2) + \boldsymbol{\tau}_1$	0.15 ± 0.03	0.22 ± 0.04
$(2,0,4) - \boldsymbol{\tau}_1$	0.14 ± 0.02	0.21 ± 0.03

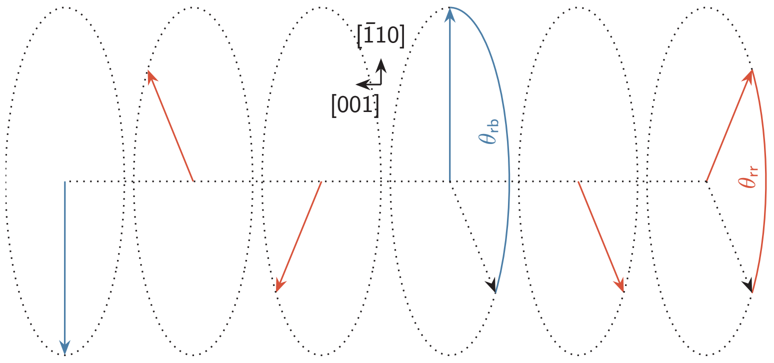

Supplementary Figure 5. **Diagram of the broken-helix order.** The ordered moment lies $\perp$ [001], and the ferromagnetically aligned Eu layers are labeled red or blue, with the blue layers constrained to point along either [$\bar{1}$10] or $-$[$\bar{1}$10]. ϕ_{rr} and ϕ_{rb} are the helical-turn angles between successive red-red and red-blue layers, respectively.

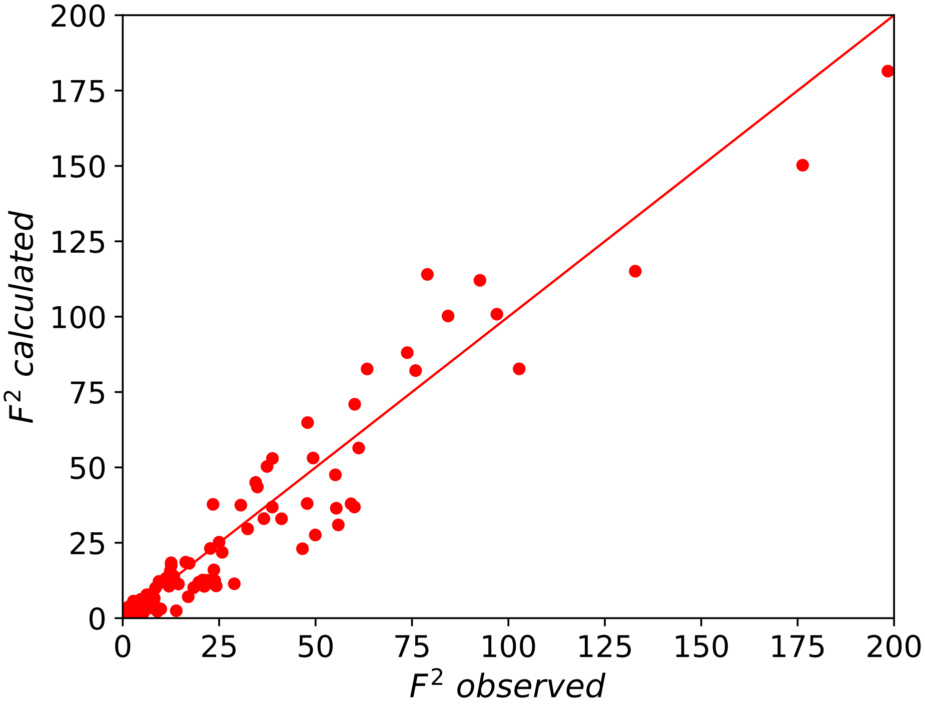

Supplementary Figure 6. **Calculated versus observed square of the magnetic structure factor F^2 for the refinement to 6 K CORELLI data using MSG $C2'2'2_1$.** The line indicates the refinement, which has a goodness of fit of $R_F = 17.2$.

179.161). As shown by the group-subgroup graph given in Supplementary Figure 18 and discussed further below, MSGs $P6_122$ and $P6_522$ are inconsistent with the stabilization of broken-helix magnetic order with MSG $C2'2'2_1$ for $T < T_{N2}$. The remaining two MSGs ($P6_12'2'$ and $P6_52'2'$) represent right-handed and left-handed chiral versions of the same pure 60°-helix order and cannot be differentiated with our data. Using either one of these MSGs and the intensity of the $(0, 0, 6) - \boldsymbol{\tau}_1$ reflection measured on TRIAX at 16 K (see Supplementary Figure 2b), we calculate that $\mu = 2.0(2)$ μ_B/Eu at 16 K.

To complement these results, we made a refinement for the $T_{N2} < T \leq T_{N1}$ magnetic phase using MSG $P6_12'2'$ and data for 20 independent Bragg peaks corresponding to $\boldsymbol{\tau}_1$ taken with CORELLI at 16.3 K. Similar to above, data corresponding to a reduced incident neutron energy bandwidth centered at $E = 50$ meV (1.54 Å) and spanning 45–55 meV were used in order to accurately perform a correction for neutron absorption using MAG2POL [2]. In agreement with our TRIAX estimation, the refinement returns $\mu = 2.5(5)$ μ_B/Eu with

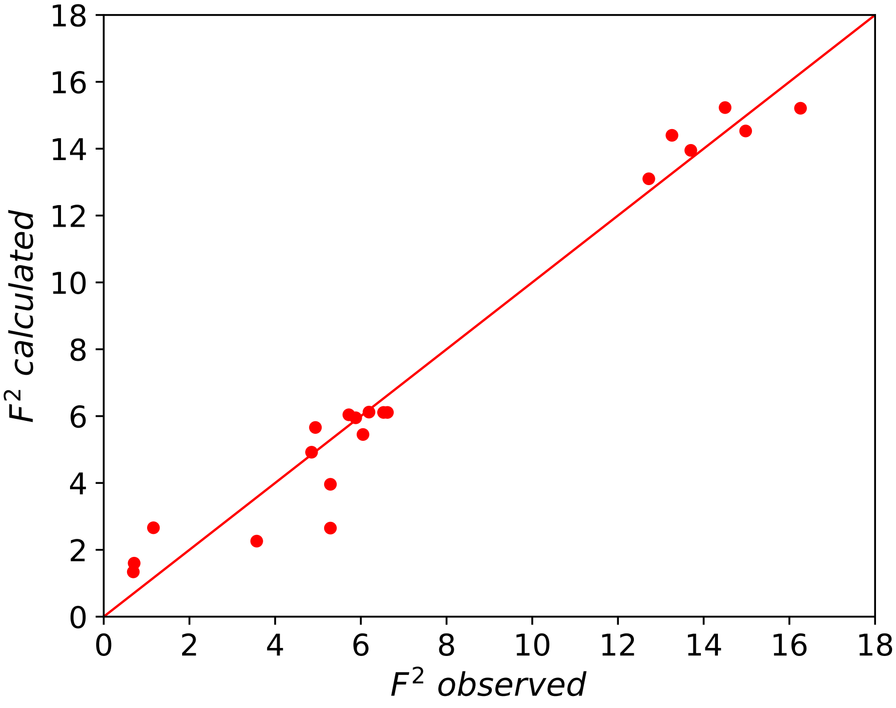

Supplementary Figure 7. **Calculated versus observed squares of the magnetic structure factor $\boldsymbol{F^2}$ for the refinement to 16.3 K CORELLI data using MSG $P6_12'2'$.** The line indicates the refinement, which has a goodness of fit of $R_F = 7.37$.

a goodness-of-fit value of $R_F = 7.37$. Supplementary Figure 7 illustrates the quality of this refinement.

In the following, we detail our determination of the magnetic phases' temperature evolution using CORELLI data.

The CORELLI data also allowed us to precisely and efficiently visualize the appearance of magnetic Bragg peaks and their positions upon cooling down to $T = 6$ K. Figure 2b displays data from magnetic order parameter measurements and Fig. 2c displays the temperature evolution of the incommensurability associated with $\boldsymbol{\tau}_1$ below T_{N1}. Large sets of data collected at 6, 16.3 and 22 K are shown in Fig. 1a and Supplementary Figure 2a. The positions of the magnetic peaks at 6 K lead to the precise determination of the two antiferromagnetic (AF) propagation vectors: (1) $\boldsymbol{\tau}_1 = (0, 0, \tau_{1z})$ with $\tau_{1z} = 0.303(1)$ corresponding to the incommensurate peaks; (2) $\boldsymbol{\tau}_2 = (0, 0, 1)$ [6] corresponding to commensurate magnetic Bragg peaks. Note that for $\boldsymbol{\tau}_2$ some nuclear and magnetic Bragg peaks overlap. CORELLI

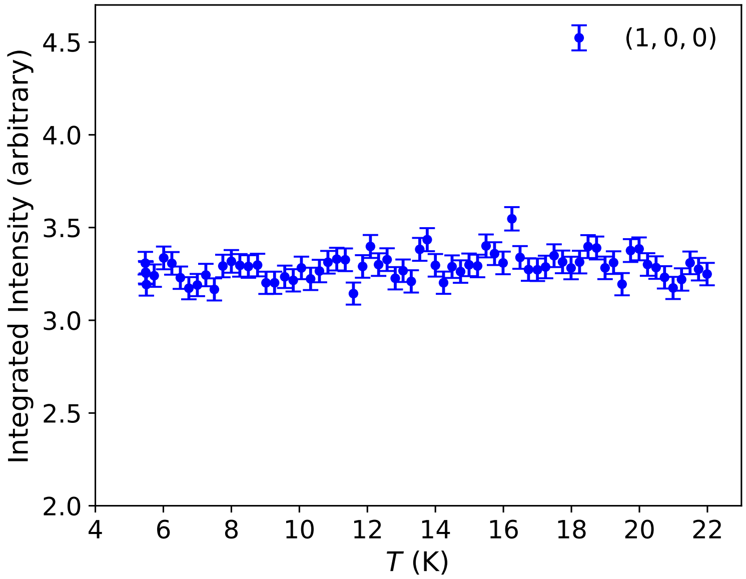

Supplementary Figure 8. **Temperature dependence of the integrated intensity of the $(1, 0, 0)$ Bragg peak measured on CORELLI .** The featureless temperature dependence between $T = 5.5$ and 22 K indicates an absence of a ferromagnetic moment pointing along $\mathbf{c}$.

data were also used to determine symmetry and ordered-moment-direction aspects of the magnetic phases. For instance, we observed the absence of magnetic Bragg peaks along the $(h00)$ direction. Supplementary Figure 8 further shows that the $(1, 0, 0)$ Bragg peak has no significant temperature dependence which, based on our simulations, indicates that $\boldsymbol{\mu}$ does not have a ferromagnetic component along $\mathbf{c}$. This information, along with the magnetization data shown in Fig. 4b and Supplementary Figure 11b support the conclusion that $\boldsymbol{\mu}$ lies in the $\mathbf{ab}$ plane below T_{N1}.

Lastly, for comparison with Fig. 2b, absorption corrected TRIAX data for the temperature dependencies of the $(0, 0, 5)$ and $(0, 0, 6 - \boldsymbol{\tau}_{1z})$ Bragg peaks are displayed in Supplementary Figure 9.

Supplementary Note 2. MAGNETIC SUSCEPTIBILITY, MAGNETIZATION, AND RESISTANCE

Supplementary Figure 10a reproduces the magnetic order parameter plots determined from neutron diffraction that are also shown in Fig. 2b. We find features corresponding to

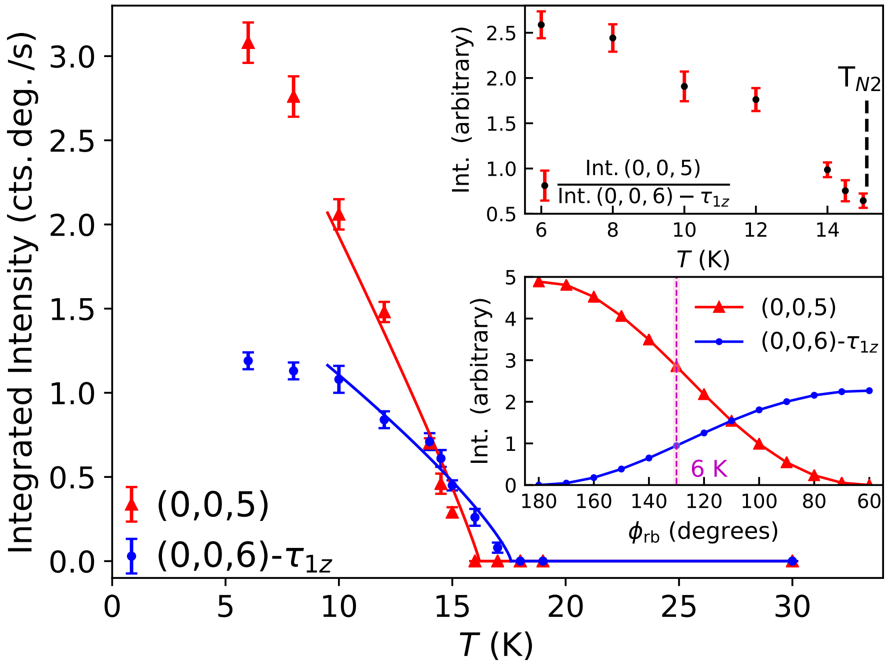

Supplementary Figure 9. **Magnetic order parameters measured on TRIAX.** The temperature evolutions of the integrated intensities of the $(0,0,5)$ and $(0,0,6-\tau_{1z})$ Bragg peaks. Lines are guides to the eye. The top right inset displays the ratio of the integrated intensities of the two peaks. The bottom right inset shows a simulation of the peaks intensities as a function of the helix turn angle $\phi_{\rm rb}$.

the two Néel temperatures $[T_{\rm N1} = 17.6(2)$ and $T_{\rm N2} = 16.2(1)$ K] in magnetization, resistance, and ^{151}Eu Mössbauer spectroscopy measurments as well. Supplementary Figure 10b. shows the temperature dependence of the magnetic susceptibility $\chi = \frac{M}{H}$ and $\frac{{\rm d}(\chi T)}{{\rm d}T}$ for an applied magnetic field of $\mu_0 H = 0.01$ T. Changes in the slope of $\chi(T)$ are evident near the dashed lines indicating $T_{\rm N1}$ and $T_{\rm N2}$.

Standard 4-wire resistance (R) measurements were made on single-crystal samples down to $T = 2$ K with a Quantum Design, Inc. Physical Property Measurement System using Pt leads attached with Epotek H20E silver epoxy. Resistance data plotted in Supplementary Figure 10c show a loss of spin-disorder scattering upon cooling through $T_{\rm N1}$ and a distinct change in slope upon further cooling through $T_{\rm N2}$.

Next, Supplementary Fig. 10d shows that the Fourier components of the hyperfine field found by ^{151}Eu Mössbauer spectroscopy measurements also show evidence for the two mag-

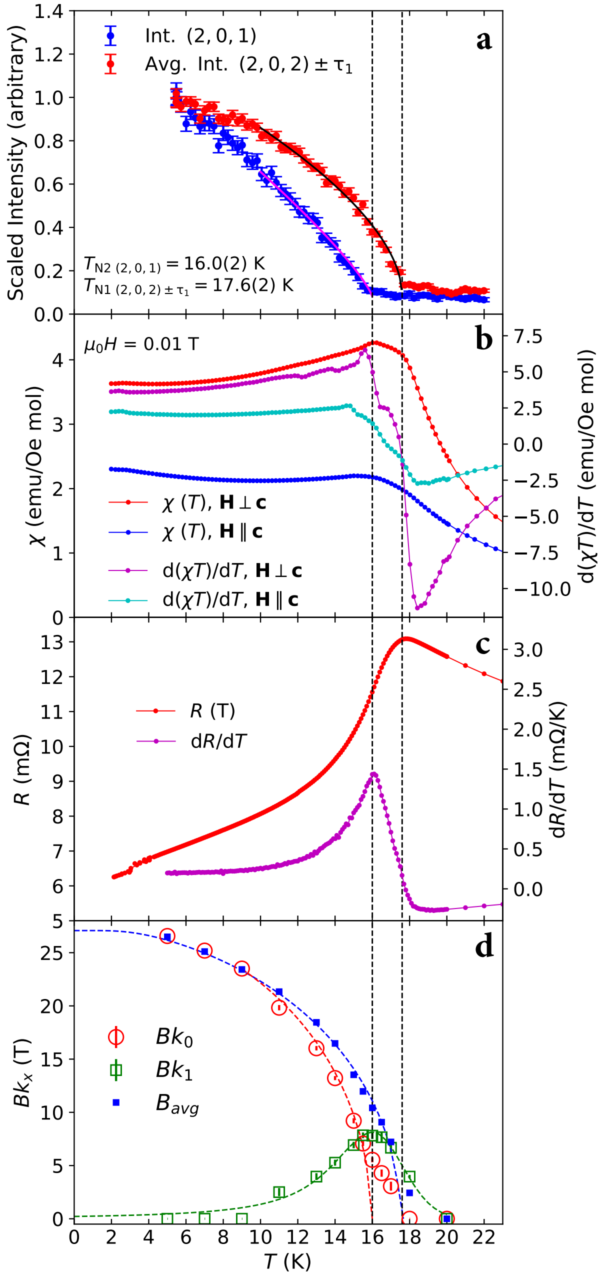

Supplementary Figure 10. **Temperature dependencies of magnetic order parameter, magnetic susceptibility, resistance, and ^{151}Eu hyperfine field.** **a**, Magnetic order parameter of the $(2,0,1)$ and $(2,0,2)\pm\tau_1$ Bragg peaks measured on CORELLI [7] scaled to be 1 at $T = 6$ K. The Néel temperatures T_{N1} and T_{N2} found by neutron diffraction are indicated by vertical dashed lines through the different panels. **b**, Magnetic susceptibility (red+blue) and its derivative with respect to temperature (magenta+cyan). **c**, Temperature evolution of the electrical resistance (red), and its derivative with respect to temperature (magenta). **d**, Temperature dependence of the Fourier components of the hyperfine field (B_{hf}) from analysis of ^{151}Eu Mössbauer spectra.

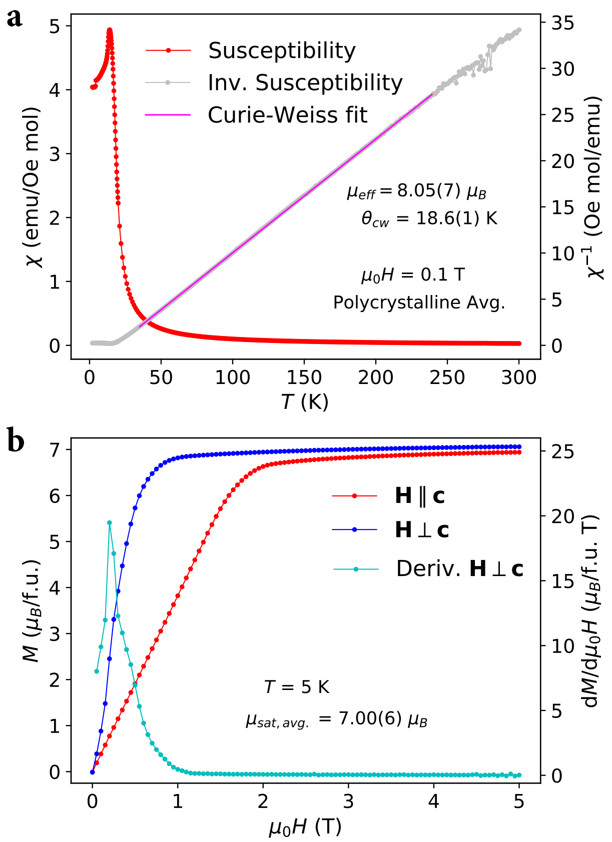

Supplementary Figure 11. **Curie-Weiss fit and magnetization versus magnetic field data at $T = 5$ K. a**, The magnetic susceptibility (red) and its inverse (grey) plotted versus temperature for an applied magnetic field of $\mu_0 H = 0.1$ T. The linear region of the inverse susceptibility is fitted with a Curie-Weiss model (fuchsia). **b**, Data from magnetization measurements performed for $\mathbf{H} \parallel \mathbf{c}$ (red) and $\mathbf{H} \perp \mathbf{c}$ (blue). The field derivative of the $\mathbf{H} \perp \mathbf{c}$ data ($\frac{\mathrm{d}M}{\mathrm{d}H}$) is also shown (cyan). Magnetic anisotropy consistent with the ordered magnetic moment lying perpendicular to $\mathbf{c}$ is evident, and a saturation magnetization of 7.00(6) μ_B/f.u. is obtained. The $\mathbf{H} \perp \mathbf{c}$ data show an inflection point at $\mu_0 H \approx 0.2$ T that is visible as a peak in $\frac{\mathrm{d}M}{\mathrm{d}H}$.

netic ordering transitions. More details concerning the Mössbauer measurements and the plots in Supplementary Figure 10d are given in a separate section below.

A Curie-Weiss fit to high-temperature $\chi(T)$ data is shown in Supplementary Figure 11a and yields an effective magnetic moment of $\mu_\mathrm{eff} = 8.05(7)\ \mu_\mathrm{B}$/Eu and a Weiss temperature of $\theta_\mathrm{CW} = 18.6(1)$ K. μ_eff is consistent with Eu^{2+}, whereas the positive value of θ_CW indicates predominantly ferromagnetic interactions and likely reflects the magnetic interactions asso-

ciated with the ferromagnetically-aligned moments within the Eu planes. The Curie-Weiss fits were performed after taking the appropriate polycrystalline average of data taken with **H** applied either perpendicular or parallel to the crystalline **c** axis.

Magnetization versus field measurements made with $\mathbf{H} \perp \mathbf{c}$ or $\mathbf{H} \parallel \mathbf{c}$ reveal magnetic anisotropy consistent with magnetic moments ordered solely within the **ab**-plane. These data appear in Fig. 4b and in Supplementary Figure 11b. A saturated moment of $\mu_{\rm sat} = 7.00(6)\ \mu_{\rm B}$/f.u. is achieved as expected for Eu^{2+}, where $M_{sat} = gS = 7\ \mu_{\rm B}$/f.u. with $g = 2$ and $S = 7/2$. g is the spectroscopic splitting factor and S is the spin. Data for $\mathbf{H} \perp \mathbf{c}$ show an inflection point at $H \approx 2$ kOe which is seen as a sharp peak in $\frac{{\rm d}M}{{\rm d}H}$. This feature may indicate a continuous crossover from helical- to fan-type AF order with increasing field [8].

Supplementary Note 3. DETAILS OF THE ^{151}Eu MÖSSBAUER STUDY

^{151}Eu Mössbauer spectroscopy measurements were carried out using a 4 GBq $^{151}SmF_3$ source driven in sine mode and calibrated using a standard ^{57}Co$\underline{\rm Rh}$/α-Fe foil. Isomer shifts are quoted relative to EuF_3 at ambient temperature. The sample was cooled in a vibration-isolated closed-cycle helium refrigerator with the sample in helium exchange gas. Spectra for different temperatures are shown in Supplementary Figure 12.

In order to determine the hyperfine field $B_{\rm hf}$, the spectra in Supplementary Figure 12 are fit as follows. Spectra for $T \lesssim 10$ K and $T \gtrsim 18$ K are fit to a sum of Lorentzian lineshapes with the positions and intensities derived from a full solution to the nuclear Hamiltonian [9]. However, spectra taken between 10 K and 18 K are fit using a model that derives a distribution of hyperfine fields from an (assumed) incommensurate magnetic structure with a sinusoidally modulated value for the ordered magnetic moment μ [10, 11]. We next describe this distribution model.

If we denote the antiferromagnetic (AF) propagation vector as **k** (instead of $\boldsymbol{\tau}$), assume that the modulation in μ along the direction of the propagation vector can be written in terms of its Fourier components, and that the hyperfine field is a linear function of μ at any given site, then the variation of $B_{\rm hf}$ with distance x along **k** can be written as:[10]

$$B_{\rm hf}(kx) = Bk_0 + \sum_{l=0}^{n} Bk_{2l+1} \sin[(2l+1)kx] \ . \qquad (1)$$

Bk_n are the odd Fourier coefficients of the field modulation and kx is a position in reciprocal

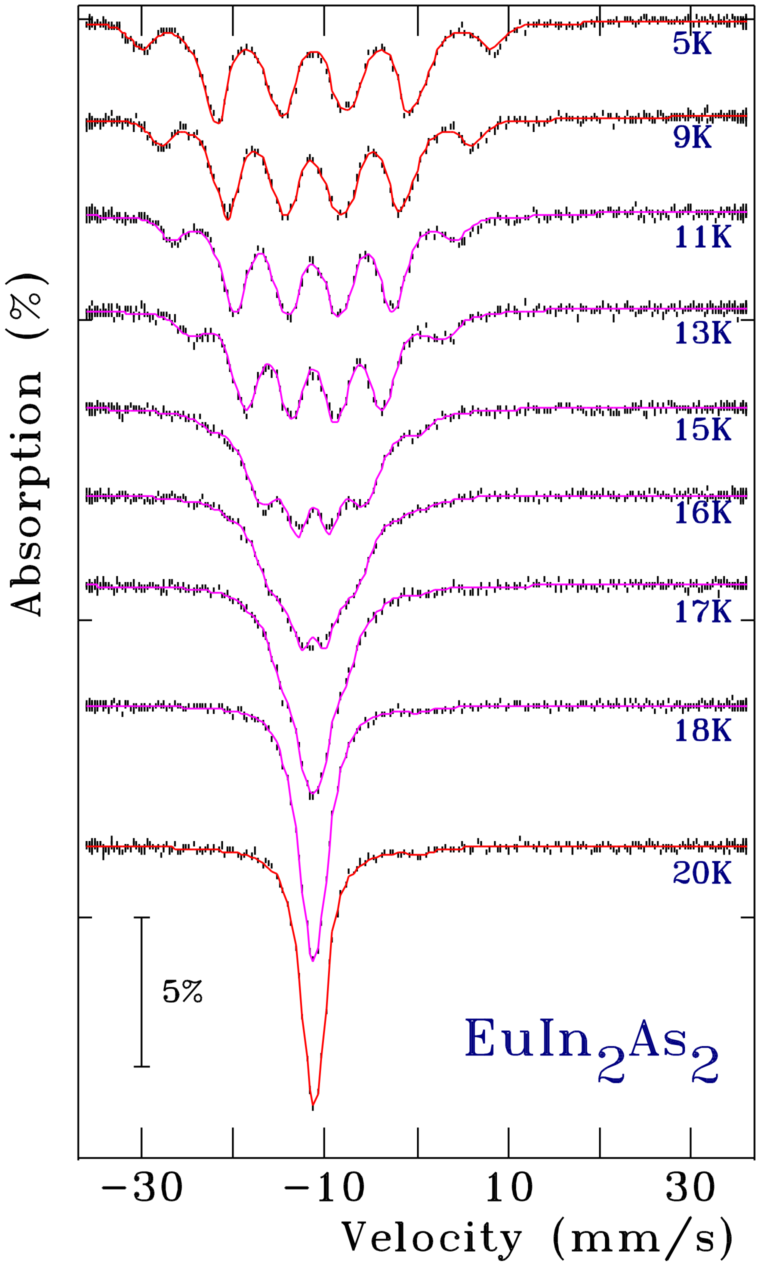

Supplementary Figure 12. **^{151}Eu Mössbauer spectra showing the evolution of the magnetic order on heating from a temperature of $T = 5$ to 20 K.** Solid lines are fits using the full Hamiltonian (red lines, 5 K, 9 K and 20 K) or the incommensurate modulated model (magenta lines). A description of the fitting procedure is given in the text.

space along the direction of $\mathbf{k}$. As $+B_{\rm hf}$ and $-B_{\rm hf}$ are indistinguishable, kx only needs to run over half the modulation period. Variations of this modeling have been used to fit spectra for EuPdSb [10] and Eu_4PdMg [12].

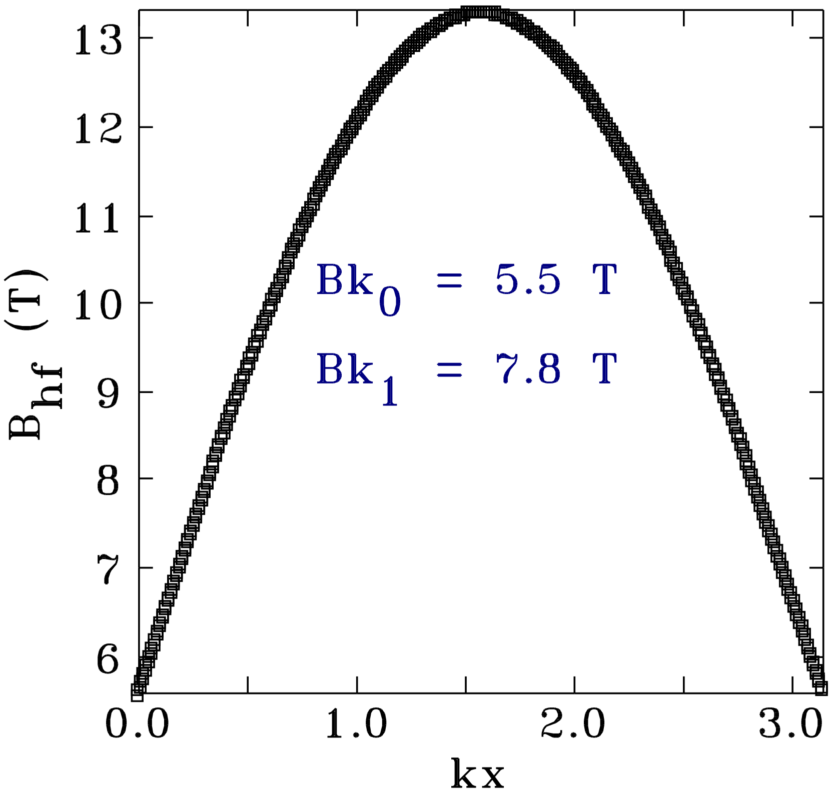

Supplementary Figure 13. **Magnitude of the hyperfine magnetic field as a function of distance along the direction of the magnetic-propagation vector for $T = 16$ K.** $kx = \pi$ corresponds to the length of the Brillouin zone along $\boldsymbol{kx}$. Values of the Fourier components Bk_0 and Bk_1 discussed in the text are indicated.

The fitting sequence is as follows: Starting with an initial set of Bk_n, the evolution of $B_{\rm hf}$ along $\mathbf{k}$ is evaluated as shown in Supplementary Figure 13. From $B_{\rm hf}$ versus kx, the histogram in Supplementary Figure 14 is constructed, and the calculated ^{151}Eu Mössbauer spectrum is obtained through a sum of magnetic patterns weighted according to the histogram. A conventional non-linear least-squares minimization routine is then used to adjust Bk_n, a uniform baseline, an overall scale factor, and the isomer shift. It is essential to note that since the modulation is explicitly assumed to be incommensurate with the crystal lattice, positions along kx do not correspond to specific positions in the chemical unit cell. Rather, they represent how $B_{\rm hf}$ is sampled by the Eu. As the periodicity of the modulation and the crystal cell are not related by a simple rational fraction, neighboring points along the kx axis will be far apart in real space.

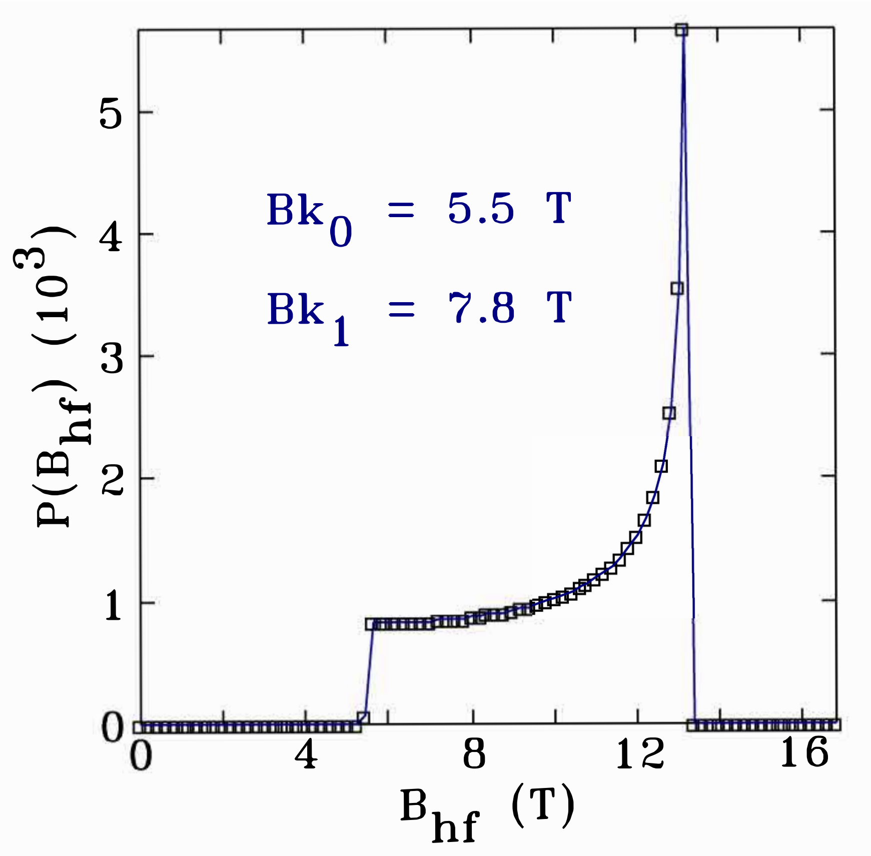

Supplementary Figure 14. **Histogram for fitting the $T = 16$ K Mossbäuer spectrum.** $B_{\rm hf}$ is the hyperfine field and $P(B_{\rm hf})$ is the probability distribution of $B_{\rm hf}$.

The room temperature ^{151}Eu Mössbauer spectrum has a single peak much like the $T =$ 20 K spectrum shown in Supplementary Figure 12. Fitting the spectrum gives an isomer shift of −11.35(2) mm/s, indicating Eu^{2+}. No trivalent impurity (within a sensitivity of 1%) was detected. The similar isomer shifts at 20 K (−11.28(1) mm/s) and 5 K (−11.20(2) mm/s) confirm that the valence does not change over the temperature range studied. The fitted $B_{\rm hf}$ of 26.46(5) T at 5 K is typical of a fully ordered Eu^{2+} moment [13], and the sharp lines are consistent with the Eu being in a single magnetic environment at 5 K. The trigonal $\bar{3}m.$ point symmetry of the Eu site leads to the requirement of an axially-symmetric electric-field-gradient tensor, so the measurements are not sensitive to rotations of $\boldsymbol{\mu}$ about $\mathbf{c}$.

Supplementary Figure 12 shows that increasing the temperature not only leads to a gradual reduction in $B_{\rm hf}$, but also to a clear increase in the linewidth. This broadening reflects a distribution of environments for the Eu, and may arise from either dynamic effects (e.g. slow paramagnetic relaxation) or from a static distribution of hyperfine fields. The

former is inconsistent with both the observed evolution of the spectral shapes and with the continued observation of well-defined magnetic Bragg peaks in the neutron diffraction data. This leaves a static distribution of hyperfine fields as the source of the line broadening. As Eu^{2+} is the only magnetic species present, a distribution in B_{hf} necessarily reflects a distribution of moment magnitudes μ. We therefore turn to the distribution model given by Supplementary Equation (1) that has been successful in describing the order in a number Eu- and Fe- based compounds [10–12, 14]. The solid lines through the spectra shown in Supplementary Figure 12 for $9 < T \leq 18$ K demonstrate that this model provides an excellent description of the spectra.

Looking at Supplementary Figure 10d, for $T = 6$ K only the uniform term is needed and B_{avg} is equivalent to Bk_0. Thus, at 6 K no modulation of μ is present, in agreement with the magnetic structure determined by neutron diffraction. However, by 11 K a weak modulation is needed to fit the spectrum: Bk_1 is no longer zero and Bk_0 starts to fall below B_{avg}. $Bk_1(T)$ peaks at ≈ 16 K and Bk_0 is only lost by 18 K. Fitting $B_{avg}(T)$ to a $J = \frac{7}{2}$ Brillouin function (appropriate for Eu^{2+}) yields 17.5(1) K, while doing the same for $Bk_0(T)$ gives 15.9(1) K, which is also where $Bk_1(T)$ peaks. These two ordering temperatures are in good agreement with $T_{\mathrm{N1}} = 17.6(2)$ K and $T_{\mathrm{N2}} = 16.2(1)$ K. determined by neutron diffraction. We also remark that $Bk_1(T)$ is finite proximate to the AF phase transitions, suggesting that the inferred modulation in μ may be related to the critical region around these phase transitions.

To summarize, our Mössbauer measurements indicate that the initial AF order that develops on cooling through 17.5 K is dominated by an incommensurate, sine-like, modulation of μ along the direction of the AF propagation vector, but a significant uniform contribution is likely also present. On further cooling, both contributions grow, but at 16 K the modulation starts to decline and the uniform term quickly dominates, until by 9 K only order with a uniform (fixed-size) value of μ remains.

Supplementary Note 4. ADDITIONAL DENSITY FUNCTIONAL THEORY RESULTS

Density functional theory (DFT) calculations including spin-orbit coupling were made after adiabatically changing ϕ_{rb}, in accordance with the magnetic space group constraints

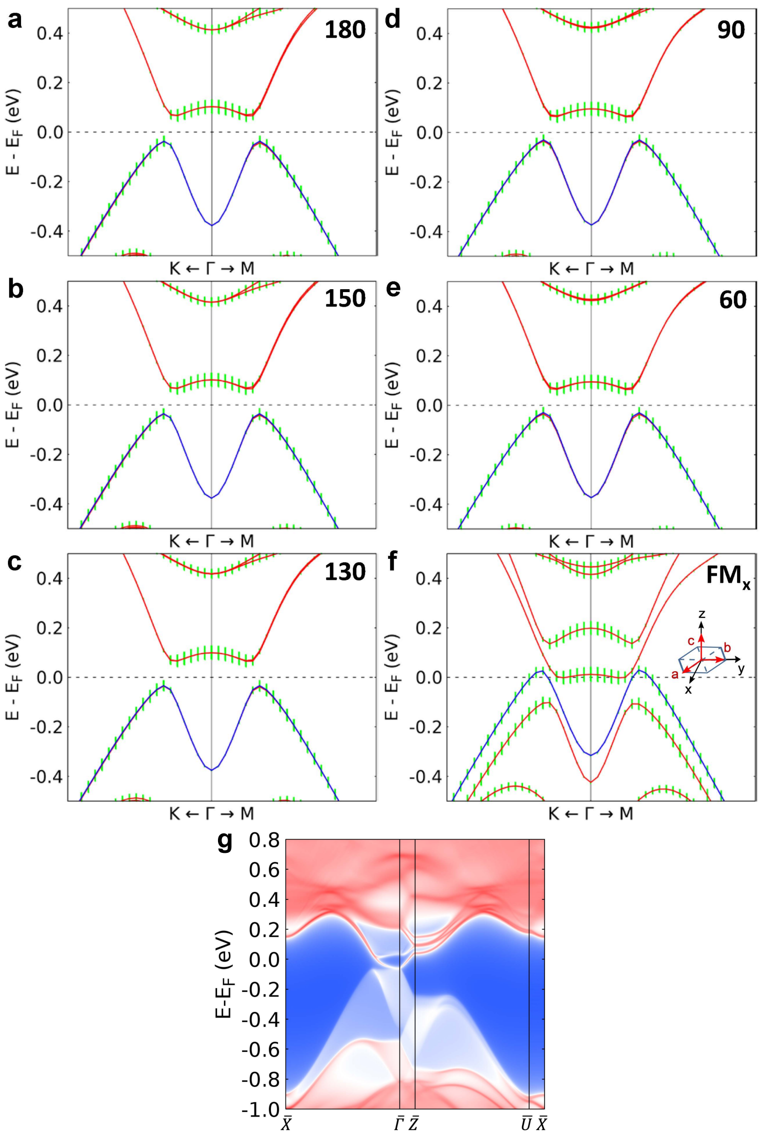

Supplementary Figure 15. **Further results from density-functional-theory calculations including spin-orbit coupling.** **a–e**, Results from calculations made after adiabatically changing the turn angle $\phi_{\rm rb}$ associated with the broken-helix ground state. The value for $\phi_{\rm rb}$ is indicated in each panel by the top-right number. The top valence band according to simple band filling is in blue and the rest of the bands are in red. Green vertical bars show As $4p_z$ orbital character and indicate band inversion near the minimal gap. **f**, Results for a ferromagnetic ground state with moments oriented along **x**. The relationship of **x** to the lattice is shown in the included diagram. **g**, Full view of the surface band structure and band gap for the (110) projected surface along high-symmetry lines for $\phi_{\rm rb} = 130°$.

$\phi_{\rm rr} + 2\phi_{\rm rb} = 180°$ and blue moments alternately pointing along $\pm[\bar{1}10]$, to values spanning the region between pure 60°-helix and A-type order shown in Fig. 2a of the main text. Results of these calculations are shown in Figs. 3b–3d and Supplementary Figures 15a–15e, where the value of $\phi_{\rm rb}$ for each panel is indicated by the number at the top right. As described in the main text, band-inversion and the electronic-band gap remain over the whole $60° \leq \phi_{\rm rb} \leq 180°$ region which implies that $\theta = \pi$ over the whole region. Supplementary Figure 15f shows that for ferromagnetic order with the ordered moment lying within the basal plane band inversion remains but degeneracy is lifted and a Fermi surface forms. A $12 \times 12 \times 3$ k-point mesh was used for the ferromagnetic calculation. Supplementary Figure 15g displays a full view of the surface band structure for the (110) projected surface along high-symmetry lines for $\phi_{\rm rb} = 130°$. This extended view complements Figs. 3e and 3f, revealing both the gap at $E_{\rm F}$ and the surface Dirac cone at $E_{\rm F} - 0.061$ eV.

Supplementary Note 5. SYMMETRY ANALYSIS OF THE MAGNETIC PHASES

In this section, we provide a detailed symmetry analysis of the magnetic space groups (MSGs) for the two observed magnetic phases: (i) the pure 60°-helix order emerging upon cooling through $T_{\rm N1} = 17.6(2)$ K, and (ii) the broken-helix order existing for $T \leq T_{\rm N2}$. The material crystallizes according to the crystallographic space group (SG) $P6_3/mmc$ (No. 194) and is built of hexagonal layers of Eu alternating with blocks of In_2As_2 along the crystallographic $\mathbf{c}$ axis [3, 4], see Fig. 1c. The unit cell contains two Eu layers separated by a distance of $c/2$. In this section, the $\mathbf{c}$ axis will also be denoted as $\hat{c} \equiv [001]$ or $\hat{z}$, and in Supplementary Figure 16a we depict our choice of $\hat{x}$, $\hat{y}$, and $\hat{z}$ axes with respect to the in-plane hexagonal directions $\hat{a} \equiv [100]$ and $\hat{b} \equiv [010]$.

The crystallographic space group $P6_3/mmc$ (No. 194) is generated by the following elements: inversion ($\mathcal{I}$), a $2\pi/3$ rotation around $\hat{z}$ ($C_{3,[001]}$), a π rotation around $\hat{z}$ followed by a $\frac{c}{2}$ translation along $\hat{z}$ ($\{C_{2,[001]} \big| 00\frac{c}{2}\}$), and a π rotation around [100]: $C_{2,[100]}$. In the paramagnetic phase, the MSG is $P6_3/mmc1'$ (No. 194.264), which is a grey group containing time-reversal [$\mathcal{T}$ (also indicated by the prime symbol)] combined with any symmetry operation of the crystallographic SG.

The two helical magnetically ordered phases are described by a magnetic unit cell that is three times larger along $\hat{c}$ than the crystallographic unit cell, $c_{\rm mag} = 3c$. As a result,

the two additional translations $\hat{c}$ and $2\hat{c}$, which are $\hat{c}_{\mathrm{mag}}/3$ and $2\hat{c}_{\mathrm{mag}}/3$ with respect to the magnetic unit cell, need to be considered. Combined with the crystallographic SG, this leads to additional symmetry operations which are non-symmorphic with respect to the magnetic unit cell. Moreover, while $\mathcal{T}$ by itself is no longer a symmetry element of the magnetically ordered phase, $\mathcal{T}$ combined with some spatial operations of the crystallographic SG are symmetry elements of the magnetic phase, and the MSG is of black-white type. The MSG is defined by the subset of operations generated by the generators of the paramagnetic MSG $P6_3/mmc1'$ combined with the translations $\{\hat{c} = \hat{c}_{\mathrm{mag}}/3, 2\hat{c} = \hat{c}_{\mathrm{mag}}/3\}$ which leave the magnetic order invariant.

We first analyze the symmetries of the pure 60°-helix phase that appears upon cooling through $T_{\mathrm{N1}} = 17.6(2)$ K as shown in Fig. 2b in the main text. The ordered magnetic moments $\boldsymbol{\mu}$ are ferromagnetically aligned in each Eu layer. As shown in Supplementary Figure 16, neighboring layers are oriented at an angle of ±60° to each other such that the structure describes a pure 60° helix propagating along $\hat{z}$. This magnetic structure repeats every six Eu-layers, leading to a tripling of the chemical unit cell. The ordered moments in layers 3 and 6 point along the high-symmetry directions $[\bar{1}10]$ and $[1\bar{1}0]$ (denoted in blue), respectively. We verified that there are 12 magnetic symmetry operations that leave the structure invariant. For a helix that rotates counterclockwise around $\hat{c}$, the corresponding MSG is $P6_12'2'$ (No. 178.159), which is generated by the set

$$\Big\{\mathbb{1}, \big\{ C_{2,[001]} \big| \, 00\frac{c_{\mathrm{mag}}}{2} \big\}, C_{3,[001]} \big\{ \mathbb{1} \big| \, 00\frac{c_{mag}}{3} \big\}, \mathcal{T}C_{2,[100]} \Big\} \ , \tag{2}$$

where $\mathbb{1}$ is the identity element. Note that a helix with opposite helicity (i.e. clockwise rotation around $\hat{c}$) is described by MSG $P6_52'2'$ (No. 179.165).

The symmetry of the pure 60°-helix order can be readily checked graphically, as shown in Supplementary Figure 16. It is important to note that a rotation around an axis different from [001] leads to a *reshuffling* of the Eu-layers as shown in Supplementary Figures 16b and 16c for $C_{2,[100]}$. The reshuffling depends on the choice of origin of the coordinate system. Here, we choose the origin to lie in Eu layer 2. Whereas the form of the generators depends on the choice of origin, the resulting MSG [$P6_12'2'$ (No. 178.159)] is independent of the origin choice.

Importantly, these MSGs contain the element $\mathcal{T}C_{2,[100]}$, which reverses an odd number of space-time dimensions, and therefore leads to a quantization of the magnetoelectric coupling

(axion) angle θ. Further, the magnetic symmetry $\mathcal{T}C_{2,[100]}$ protects exotic unpinned gapless surface Dirac states on the $(2\bar{1}0)$ surface [15]. This MSG contains five more $2' = \mathcal{T}C_2 =$ elements that protect unpinned Dirac cones on five more surfaces. These are the surfaces $(\bar{1}20)$ and (110), which are related to $(2\bar{1}0)$ by $C_{3,[001]}$ and $C^2_{3,[001]}$ rotations, respectively, as well as the surfaces (100), (010) and $(\bar{1}10)$, which are related to each other by $C_{3,[001]}$ rotations. A list of the $2'$ symmetry axes and surfaces with gapless Dirac cones for the various magnetic phases is given in Fig. 4c.

In the broken-helix state occurring for $T \leq T_{\rm N2} = 16.2(1)$ K, the (blue) ordered magnetic moments in Eu-layers 3 and 6 continue to point along the high-symmetry directions $[\bar{1}10]$ and $[1\bar{1}0]$, respectively. The red ordered moments (red layers), however, are not oriented at $\pm 60^\circ$ with respect to the layer immediately above or below as shown in Figs. 1d, 2a, and Supplementary Figure 5. As a result, $C_{3,[001]}$, which is a symmetry element for the 60°-helix order, is lost in the broken-helix state. The broken-helix phase is described by MSG $C2'2'2_1$ (No. 20.33), which is generated by the element set

$$\{\mathbb{1}, \{C_{2,[001]}|\, 00\tfrac{c_{\rm mag}}{2}\}, \mathcal{T}C_{2,[110]}\}\ . \qquad (3)$$

Here, we have set the origin to lie in Eu layer 3 (blue). Since the MSG contains the element $\mathcal{T}C_{2[110]}$, θ is still quantized. The [110] direction is perpendicular to the high-symmetry direction of the blue moments. Note that, in contrast to the 60°-helix order, here we only have two $2'$ operations, one $2'$ axis along [110] and the other along $[1\bar{1}0]$. This has profound consequences on the location of the unpinned gapless surface Dirac cones protected by $2'$ [15]: they appear only on (110) and $(\bar{1}10)$ surfaces for broken-helix magnetic order, whereas they can also be found on four other surfaces [(100), (010), $(\bar{2}10)$ and $(\bar{1}20)$] for the pure 60° helix state.

We next consider A-type antiferromagnetic order similar to that discussed in Supplementary Reference [16] for MSG $Cmcm$ (No. 63.457) and ordered moments lying along [100]. Here, the ordered Eu magnetic moments are again ferromagnetically-aligned in each Eu-layer, but the stacking along $\hat{c}$ is simply antiferromagnetic. In contrast to Supplementary Reference [16], we consider moments lying parallel to the high-symmetry $[\bar{1}10]$ directions and use MSG $Cm'c'm$. The A-type order is smoothly connected to the experimentally determined broken-helix and pure 60°-helix orders by varying the helical-turn angles $\phi_{\rm rb}$ and $\phi_{\rm rr}$, where $\phi_{\rm rr} = \pi - 2\phi_{\rm rb}$ as shown in Fig. 2a of the main text. $|\phi_{\rm rb}| = |\phi_{\rm rr}| = \pi$ for the

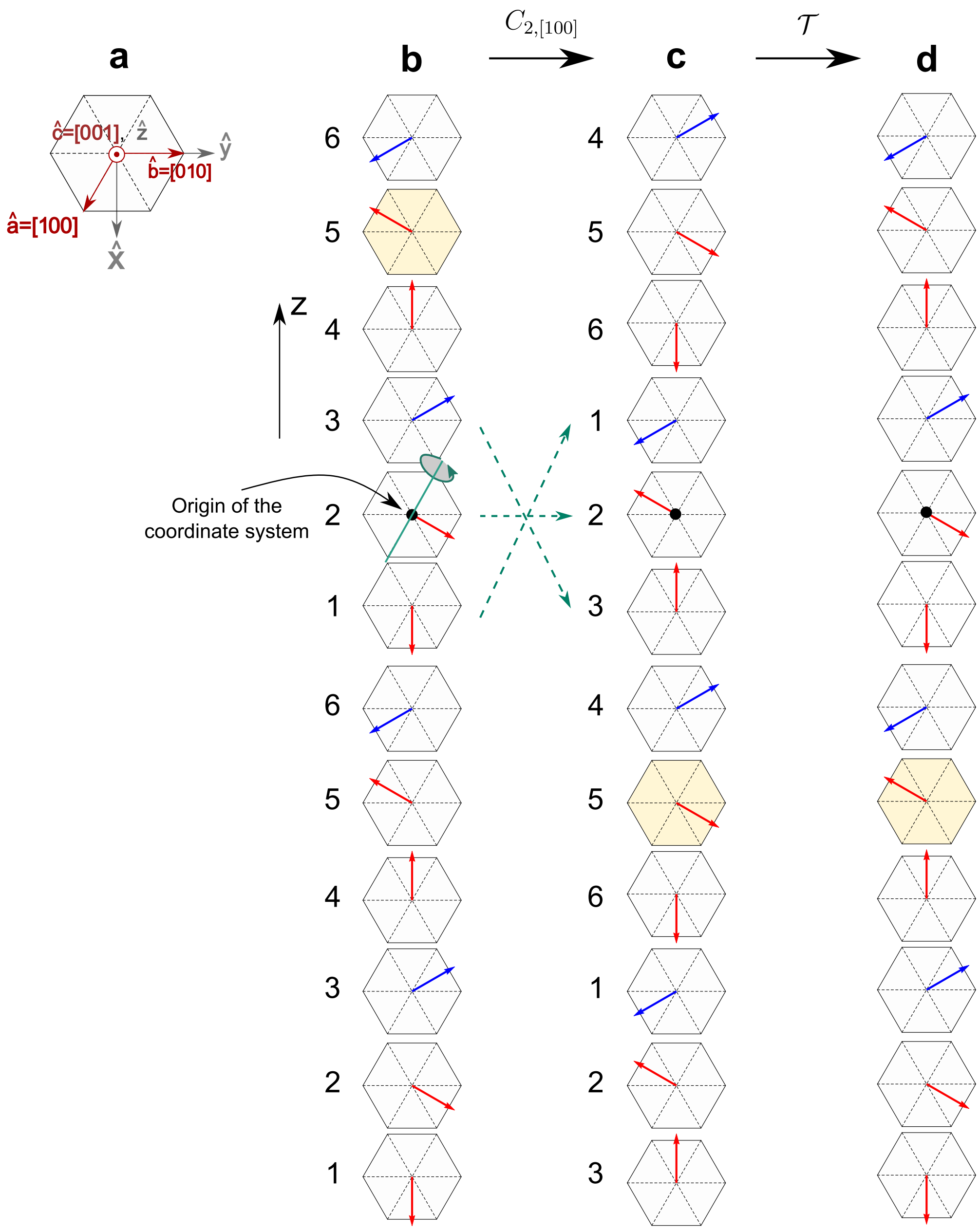

Supplementary Figure 16. **Action of $\mathcal{T}C_{2,[100]}$ on pure 60°-helix order.** **a**, Crystallographic $\hat{a}$, $\hat{b}$ and $\hat{c}$ axes for hexagonal $EuIn_2As_2$ together with our choice of $\hat{x}$, $\hat{y}$ and $\hat{z}$ axes. **b**, 60°-helix order where two magnetic unit cells are shown. **c, d**, The remaining panels show the action of $\mathcal{T}C_{2,[100]}$ on the 60°-helix phase: a π rotation around the [100] axis (c) followed by a time-reversal operation (d) leaves the initial structure invariant. Therefore, $\mathcal{T}C_{2,[100]}$ is a symmetry operation of the pure 60°-helix order. One of the Eu-layers is highlighted to facilitate the visualization of the action of each of the space-time transformations. Note that we set the origin of the coordinate system in the second Eu layer.

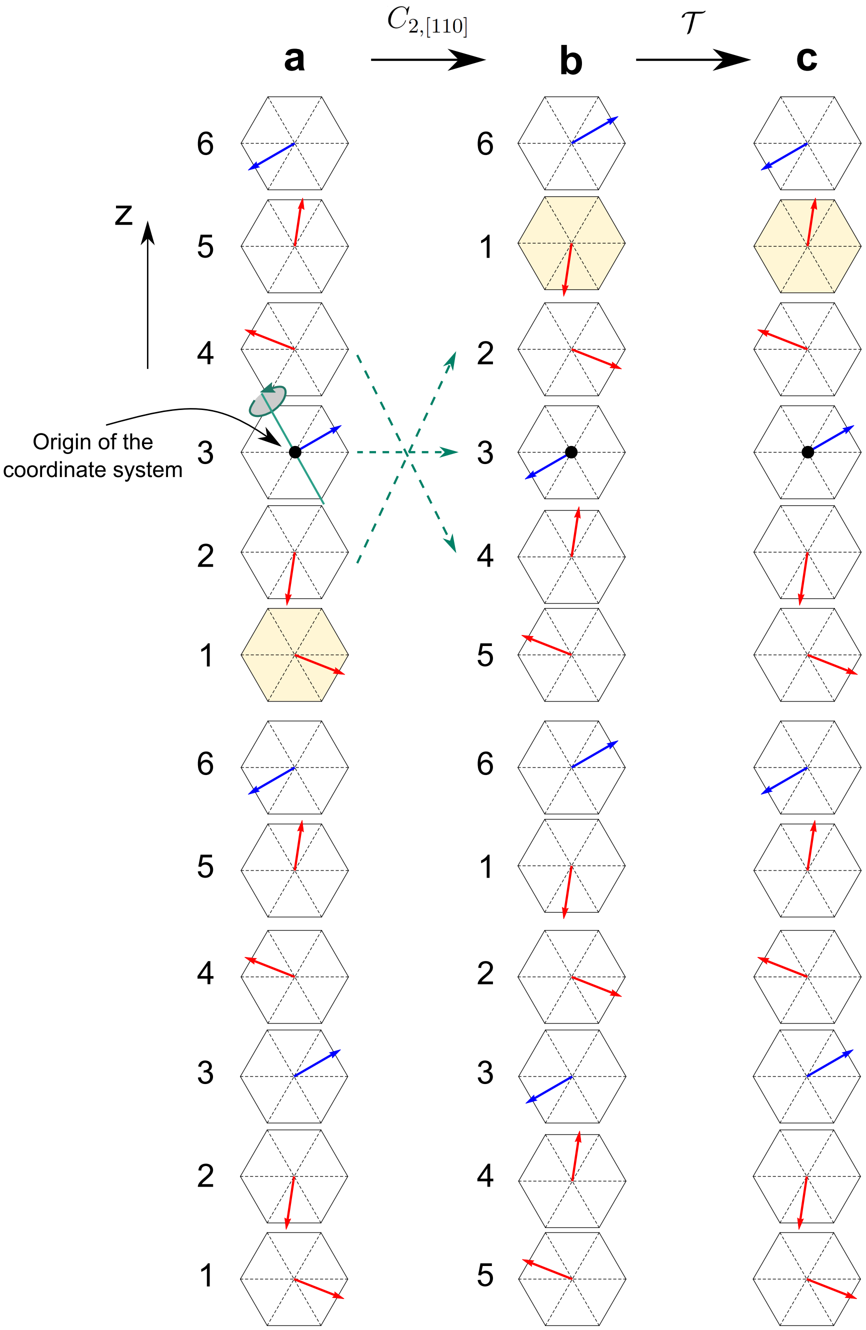

Supplementary Figure 17. **Action of $\mathcal{T}C_{2,[110]}$ on broken-helix order.** **a**, Initial magnetic structure, where two magnetic unit cells are shown. **b, c**, A π rotation around the [110] axis (b) followed by a time-reversal operation (c) leaves the initial structure invariant. Therefore, $\mathcal{T}C_{2,[110]}$ is a symmetry operation of the broken-helix structure. Similar to Supplementary Figure 16, one of the Eu-layers is highlighted to facilitate the visualization of the action of each of the space-time transformations. Here, the origin of the coordinate system is set in the third Eu layer.

A-type state. Since the crystallographic unit cell contains two Eu layers, the magnetic unit cell is equal to the crystallographic one in this case. The MSG of A-type order with ordered moments along [1$\bar{1}$0] is $Cm'c'm$ (No. 63.462), which is generated by

$$\{\mathbb{1}, \mathcal{I}, \{C_{2,[001]}|\, 00\frac{c}{2}\}, \mathcal{T}C_{2,[110]}\}\,. \tag{4}$$

We find that gapless surface Dirac cones, which can be shifted away from the center of the surface Brillouin zone, occur on the (110) and ($\bar{1}$10) surfaces. In contrast to the 60°-helix order and the broken-helix order, here the Dirac cones are constrained to occur along the $\bar{\Gamma}$-$\bar{\mathrm{X}}$ direction of the surface Brillouin zones due to the mirror symmetry $\{m_{[001]}|\, 00\frac{c}{2}\}$ that is preserved on both surfaces.

Importantly, due to the presence of $\mathcal{I}$ the energy bands have well-defined parity. This allows us to easily infer the value of θ by analyzing the parity-based $\mathbb{Z}_4$ invariant, as explained in the main text. By our DFT calculations, we find $\mathbb{Z}_4 = 2$ for the A-type state, which corresponds to $\theta = \pi$ and an axion insulator (AXI) phase. As our DFT results show that the band gap never closes when we continuously change the angles ϕ_{rb} and ϕ_{rr} from $|\phi_{\mathrm{rr}}| = |\phi_{\mathrm{rb}}| = \pi$ to their experimentally determined values in the broken- and 60°-helix states, we conclude that both the broken- and pure 60°-helix states also have $\theta = \pi$ and are therefore AXIs. Note that while $\mathcal{I}$ is absent in the experimentally determined helical structures, the combination $\mathcal{T}C_2 = 2'$ guarantees quantization of θ, as it reverses an odd number of space-time coordinates. Finally, we note that, as discussed in Supplementary Reference [16], A-type order with ordered moments along [100] or [010] is described by MSG $Cmcm$ (No. 63.457), whereas A-type order with moments along [001] is described by MSG $P6_{3'}/m'm'c$ (No. 194.268).

Several magnetic phase transitions occur in $\mathrm{EuIn_2As_2}$ upon cooling, which in Landau theory for second-order phase transitions may be related to transitions from a symmetry group to one of its subgroups. Starting from the high-temperature paramagnetic phase, the compound undergoes a transition to pure 60°-helix order below $T_{\mathrm{N1}} = 17.6(2)$ K, followed by a second transition to broken-helix order below $T_{\mathrm{N2}} = 16.2(1)$ K. From the point of view of group-subgroup relations, there are three different paths the system could follow between the high-temperature MSG $P6_3/mmc1'$ and the low-temperature MSG $C2'2'2_1$ as shown in Supplementary Figure 18. The left path in Supplementary Figure 18 corresponds to the Eu moments first aligning ferromagnetically along [001], followed by a transition to A-type

order, and finally by a third transition to the broken-helix phase. This path is ruled out by our neutron diffraction data, since no out-of-plane component of the ordered magnetic moment is observed (see Supplementary Figure 8) and only two ordering transitions were seen.

The other two paths in Supplementary Figure 18 correspond to transitions from the paramagnetic state to pure 60°-helix order and, finally, to the broken-helix state. Based on our experiments, both of these routes are possible with the only difference between the two cases being the handedness (chirality) of the pure 60° helix phases: the moments rotate clockwise around [001] for the center path and counterclockwise around [001] for the right path. It is important to note that the handedness of the helical states cannot be resolved from our data, and it is likely that domains with different handedness coexist in the sample. Also, note that the broken-helix state also exhibits a handedness, yet both right- and left-handed chiralities are described by MSG $C2'2'2_1$.

Polarizing the Eu moments along a specific direction via applying an external magnetic field offers tuning of the topological properties of the material. As shown in Fig. 4b and Supplementary Figure 11b, at $T = 5$ K a magnetic field of $\mu_0 H \approx 1$ to 2 T aligns the Eu magnetic moments parallel to the field, which corresponds to a field-polarized state. Below, we study the symmetry and topological consequences of a field-polarized state by considering three orientations for the external field.

We first consider a magnetic field along the high-symmetry direction $[\bar{1}10]$, which is the direction that the blue Eu ordered magnetic moments lie along. The resulting field-polarized state with all moments aligned along $[\bar{1}10]$ is characterized by MSG $Cm'cm'$ (No. 63.464), which is generated by

$$\{\mathbb{1}, \mathcal{I}, \mathcal{T}\{C_{2,[001]}|\,00\frac{c}{2}\}, \mathcal{T}C_{2,[110]}\}\ . \tag{5}$$

In this phase, there is both $\mathcal{T}C_{2,[110]} \equiv 2'_{[110]}$ and $\mathcal{T}\{C_{2,[001]}|\,00\frac{c}{2}\} \equiv \{2'_{[001]}|\,00\frac{c}{2}\}$. While the former protects a gapless Dirac cone on the (110) surface, the latter is a screw axis that is naturally broken on the (001) surface. Therefore, (110) is the only surface hosting an exotic surface state in this polarized phase. Importantly, because the mirror symmetry $\{m_{[\bar{1}10]}|\,00\frac{c}{2}\}$ is preserved in this surface, the Dirac cone occurs along the $\bar{\Gamma}$-$\bar{\mathrm{Z}}$ direction in the surface Brillouin zone.

Another possibility is to align the Eu moments along [100]. In this case, the MSG is

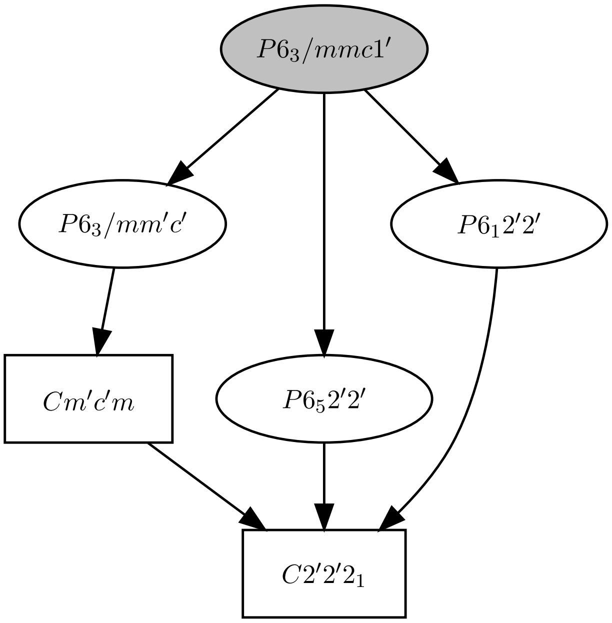

Supplementary Figure 18. **Magnetic subgroup tree.** Magnetic group–subgroup tree connecting the paramagnetic magnetic space group (MSG) $P6_3/mmc1'$ to MSG $C2'2'2_1$, which corresponds to the broken-helix order. The middle and right paths reach the low-temperature phase via intermediate 60° helical order propagating along **c** with either counterclockwise (right path) or clockwise (center path) chirality. Both of these paths agree with the experimental results. The left path traverses A-type order ($Cm'c'm$) with moments along $[\bar{1}10]$ (or $[1\bar{1}0]$), which is not experimentally realized. This diagram is taken from Supplementary Reference [1].

$Cmc'm'$ (No. 63.463), with generators

$$\left\{\mathbb{1}, \mathcal{I}, C_{2,[100]}, \mathcal{T}\left\{C_{2,[001]}\middle|\, 00\frac{c}{2}\right\}\right\} \ . \tag{6}$$

The combinations of the generators in Supplementary Equation (6) result in two $2'$ operations: $\left\{2'_{[001]}\middle|\,00\frac{c}{2}\right\}$ and $\left\{2'_{[120]}\middle|\,00\frac{c}{2}\right\}$. As above, the screw operation $\left\{2'_{[001]}\middle|\,00\frac{c}{2}\right\}$ is naturally broken on the (001) surface. Therefore, here we only have a Dirac surface state on the (010) surface, which is pinned along the $\bar{\Gamma}$-$\bar{\text{Z}}$ direction due to an additional $m_{[100]}$ mirror symmetry.

If we apply a magnetic field perpendicular to the Eu layers so that the moments align parallel to $\hat{c}$, we obtain a magnetic state belonging to MSG $P6_3/mm'c'$ (No. 194.270). The generators of this group are

$$\{\mathbb{1}, \mathcal{I}, C_{3,[001]}, \left\{C_{2,[001]}\middle|\, 00\frac{c}{2}\right\}, \mathcal{T}C_{2,[100]}\} \, . \tag{7}$$

	MSG	X_{BS}
Perfect helix	$P6_12'2'$ (No. 178.159)	1
Broken helix	$C2'2'2_1$ (No. 20.33)	1
A-type $\parallel$ [$\bar{1}$10]	$Cm'c'm$ (No. 63.462)	2
Field-pol. $\parallel$ [$\bar{1}$10]	$Cm'cm'$ (No. 63.464)	$(2, 2)$
Field-pol. $\parallel$ [100] (or [010])	$Cmc'm'$ (No. 63.464)	$(2, 2)$
Field-pol. $\parallel$ [001]	$P6_3/mm'c'$ (No. 194.270)	$(3, 6)$

Supplementary Table IV. **Symmetry-based indicators of band topology.** Data taken from Supplementary Reference 17 for all of the magnetic phases described in this section.

They give rise to 24 symmetry operations, among which six are combinations of $\mathcal{T}$ and C_2 rotations. These $2'$ operations can be grouped into two different classes: one class contains the element $2'_{[100]}$ and those related to it by $C_{3,[001]}$. As a consequence, Dirac surface states emerge on the surfaces ($2\bar{1}0$), ($1\bar{2}0$) and (110). The second class contains the element $\left\{2'_{[120]}\middle| 00\frac{c}{2}\right\}$ and those related to it by $C_{3,[001]}$. Note that the direction of these $2'$ operations are normal to the side faces of the hexagonal crystallographic unit cell, and they protect Dirac surface states on the (100), (010) and ($1\bar{1}0$) surfaces.

Note that the surfaces hosting exotic states are the same as those for pure 60°-helix order. The difference here is that the Dirac cones are no longer completely unpinned, but are constrained to occur along $\bar{\Gamma}$-$\bar{\mathrm{X}}$ because of the additional mirror symmetry $\left\{m_{[001]}\middle| 00\frac{c}{2}\right\}$ resulting from the combination of inversion $\mathcal{I}$ and the screw operation $\left\{C_{2,[001]}\middle| 00\frac{c}{2}\right\}$.

It becomes evident from the previous analysis that we can use an external magnetic field to either induce or destroy topological states on different surfaces of $EuIn_2As_2$. For instance, applying a magnetic field along [$\bar{1}$10] inside the pure 60°-helix phase gaps out Dirac cones on (100), (010), ($2\bar{1}0$) and ($1\bar{2}0$) surfaces and pins the gapless Dirac cones on (110) and ($1\bar{1}0$) to the $\bar{\Gamma}$-$\bar{\mathrm{Z}}$ direction in the surface Brillouin zone. If, on the other hand, a field parallel to [001] is applied in the pure 60°-helix phase, the Dirac cones remain gapless on all six surfaces but are now all constrained to lie along the $\bar{\Gamma}$-$\bar{\mathrm{X}}$ direction.

In Supplementary Table IV, we show the symmetry-based indicators X_{BS} of topology for the magnetic helical phases and the field-polarized phases. While symmetry indicators are absent for the MSGs describing the two helical phases, since $X_{BS} = 1$ means that there exist

no symmetry indicators, the MSGs of A-type order and the field-polarized phases feature differing symmetry-based indicators.

---

[1] Perez-Mato, J. M. *et al.* Symmetry-based computational tools for magnetic crystallography. *Annual Review of Materials Research* **45**, 217-248 (2015).

[2] Qureshi, N. *Mag2Pol*: A program for the analysis of spherical neutron polarimetry, flipping ratio and integrated intensity data. *Journal of Applied Crystallography* **52**, 175-185 (2019).

[3] Goforth, A. M., Klavins, P., Fettinger, J. C. & Kauzlarich, S. M. Magnetic properties and negative colossal magnetoresistance of the rare earth zintl phase $EuIn_2As_2$. *Inorganic Chemistry* **47**, 11048-11056 (2008).

[4] Rosa, P. F. S. *et al.* Electron spin resonance of the intermetallic antiferromagnet $EuIn_2As_2$. *Physical Review B* **86**, 094408 (2012).

[5] Busing, W. R. & Levy, H. A. High-speed computation of the absorption correction for single-crystal diffraction measurements. *Acta Crystallographica* **10**, 180-182 (1957).

[6] $\boldsymbol{\tau}_2 = (0,0,1)$ is equivalent to $\boldsymbol{\tau}_2 = (0,0,0)$ for $P6_3/mmc$. We use $\boldsymbol{\tau}_2 = (0,0,1)$ to facilitate presentation of the diffraction data.

[7] Ye, F., Liu, Y., Whitfield, R., Osborn, R. & Rosenkranz, S. Implementation of cross correlation for energy discrimination on the time-of-flight spectrometer CORELLI. *Journal of Applied Crystallography* **51**, 315-322 (2018).

[8] Johnston, D. C. Magnetic structure and magnetization of helical antiferromagnets in high magnetic fields perpendicular to the helix axis at zero temperature. *Physical Review B* **96**, 104405 (2017).

[9] Voyer, C. J. & Ryan, D. H. A complete solution to the Mössbauer problem all in one place. *Hyperfine Interact.* **170**, 91 (2006).

[10] Bonville, P., Hodges, J., Shirakawa, M., Kasaya, M. & Schmitt, D. Incommensurate modulated magnetic structure in orthorhombic EuPdSb. *The European Physical Journal B-Condensed Matter and Complex Systems* **21**, 349-355 (2001).

[11] Maurya, A., Bonville, P., Thamizhavel, A. & Dhar, S. $EuNiGe_3$, an anisotropic antiferromagnet. *Journal of Physics: Condensed Matter* **26**, 216001 (2014).

[12] Ryan, D. H. *et al.* Modulated ferromagnetic ordering and the magnetocaloric response of

Eu_4PdMg. *Journal of Applied Physics* **117**, 17D108 (2015).

[13] Ryan, D. H. & Cadogan, J. M. ^{151}Eu hyperfine fields, isomer shifts and moments in Eu-based EuT_2X_2 intermetallic compounds. *Hyperfine Interactions* **226**, 243-255 (2014).

[14] Duttine, M. *et al.* Modulated magnetic structure in ^{57}Fe doped orthorhombic $YbMnO_3$: A Mössbauer study. *AIP Advances* **9**, 035008 (2019).

[15] Fang, C. & Fu, L. New classes of three-dimensional topological crystalline insulators: Non-symmorphic and magnetic. *Physical Review B* **91**, 161105 (2015).

[16] Xu, Y., Song, Z., Wang, Z., Weng, H. & Dai, X. Higher-order topology of the axion insulator $EuIn_2As_2$. *Physical Review Letters* **122**, 256402 (2019).

[17] Watanabe, H., Po, H. C. & Vishwanath, A. Structure and topology of band structures in the 1651 magnetic space groups. *Science Advances* **4**, (2018).
